# Cardiovascular Comorbidity in Obstructive Sleep Apnea: The “Excessive Daytime Sleepiness” Phenotype Versus Comorbid Insomnia and Sleep Apnea (COMISA)

**DOI:** 10.3390/diagnostics16213628

**Published:** 2026-11-03

**Authors:** Nicoleta Stefania Balta (Motoc), Teodora Alexescu, Ana Maria Sandu, Andrada Urda-Cimpean, Andreea-Olivia Popa, Doina Adina Todea

**Affiliations:** 1 Department of Pulmonology, Faculty of Medicine, “Iuliu Hațieganu” University of Medicine and Pharmacy, 400012 Cluj-Napoca, Romania; nicoleta.motoc@umfcluj.ro (N.S.B.); doina_adina@yahoo.com (D.A.T.); 2 Department of Internal Medicine, Faculty of Medicine, “Iuliu Hațieganu” University of Medicine and Pharmacy, 400012 Cluj-Napoca, Romania; 3 Faculty of Medicine, “Iuliu Hațieganu” University of Medicine and Pharmacy, 400012 Cluj-Napoca, Romania; anamariasandu10@gmail.com (A.M.S.); andreeaolivia19@gmail.com (A.-O.P.); 4 Department of Medical Informatics and Biostatistics, “Iuliu Hațieganu” University of Medicine and Pharmacy, 400012 Cluj-Napoca, Romania; aurda@umfcluj.ro

**Keywords:** obstructive sleep apnea, excessive daytime sleepiness, COMISA, insomnia, obesity, dyslipidemia, cardiometabolic risk, phenotype

## Abstract

**Background/Objectives:** Excessive daytime sleepiness (EDS) and comorbid insomnia and sleep apnea (COMISA) are common clinical phenotypes of obstructive sleep apnea (OSA). We compared their cardiovascular and metabolic comorbidity. **Methods:** Cross-sectional, single-center retrospective analysis of adults with polygraphy-confirmed OSA (apnea–hypopnea index ≥ 5 events/h). The EDS phenotype was defined as an Epworth Sleepiness Scale score ≥ 10 without insomnia (*n* = 54), and COMISA as OSA with clinically reported insomnia (*n* = 45). Multivariable logistic regression identified factors independently associated with phenotype. **Results:** OSA severity and the prevalence of any cardiovascular disease (90.7% vs. 86.7%) were similar. In the adjusted model, higher BMI (aOR 0.92 per kg/m^2^) and cardiac arrhythmias (aOR 0.20) were associated with lower odds of COMISA, and dyslipidemia with higher odds (aOR 2.51). **Conclusions:** Both phenotypes carry a high cardiovascular burden, but they seem to differ in metabolic profile: severe obesity and an arrhythmic tendency in sleepy OSA, and dyslipidemia in COMISA.

## 1. Introduction

Obstructive sleep apnea (OSA) is a heterogeneous disorder whose clinical expression extends well beyond the apnea–hypopnea index (AHI). Itsassociation with hypertension, coronary artery disease, heart failure and arrhythmias is well established [1,2]. Two clinical phenotypes are frequently met in the everyday sleep-medicine practice: the symptomatic, “excessive daytime sleepiness” patient, in whom daytime hypersomnolence is the leading complaint [3,4], and the patient with comorbid insomnia and sleep apnea (COMISA), in whom difficulty initiating or maintaining sleep coexists with sleep-disordered breathing [5,6]. These phenotypes are not merely descriptive. Cluster-analytic and prospective cohort studies indicate that symptom subtypes of OSA differ in their association with incident cardiovascular disease [7,8,9].

The excessive daytime sleepiness phenotype has been linked to a higher incidence of cardiovascular events [8,9,10], possibly reflecting greater sympathetic activation, intermittent hypoxemia and a stronger inflammatory and metabolic footprint [11,12,13]. Measures of hypoxic burden that capture the depth and duration of desaturation have emerged as stronger predictors of cardiovascular outcome than the AHI alone [14,15,16]. COMISA, in turn, has been associated with increased all-cause mortality [17], with prevalent and incident cardiovascular and cerebrovascular disease [18,19,20], and with a distinct physiological and cardiometabolic profile [21,22,23,24].

Obesity, dyslipidemia and type 2 diabetes mellitus are closely intertwined with OSA and are central to its cardiovascular risk. Recent data suggest that the metabolic signature of OSA may itself depend on the symptom phenotype [25]. Despite this, direct head-to-head comparisons of the cardiometabolic burden of the EDS and COMISA phenotypes within a single clinical population remain scarce, particularly in Central and Eastern European cohorts, and it remains uncertain whether phenotype-guided management modifies cardiovascular risk [26,27].

We therefore performed a cross-sectional comparison of the prevalence of cardiovascular and metabolic disease in patients with EDS and COMISA in a single tertiary pulmonology sleep laboratory.

## 2. Materials and Methods

### 2.1. Study Design and Participants

**Figure 1:**
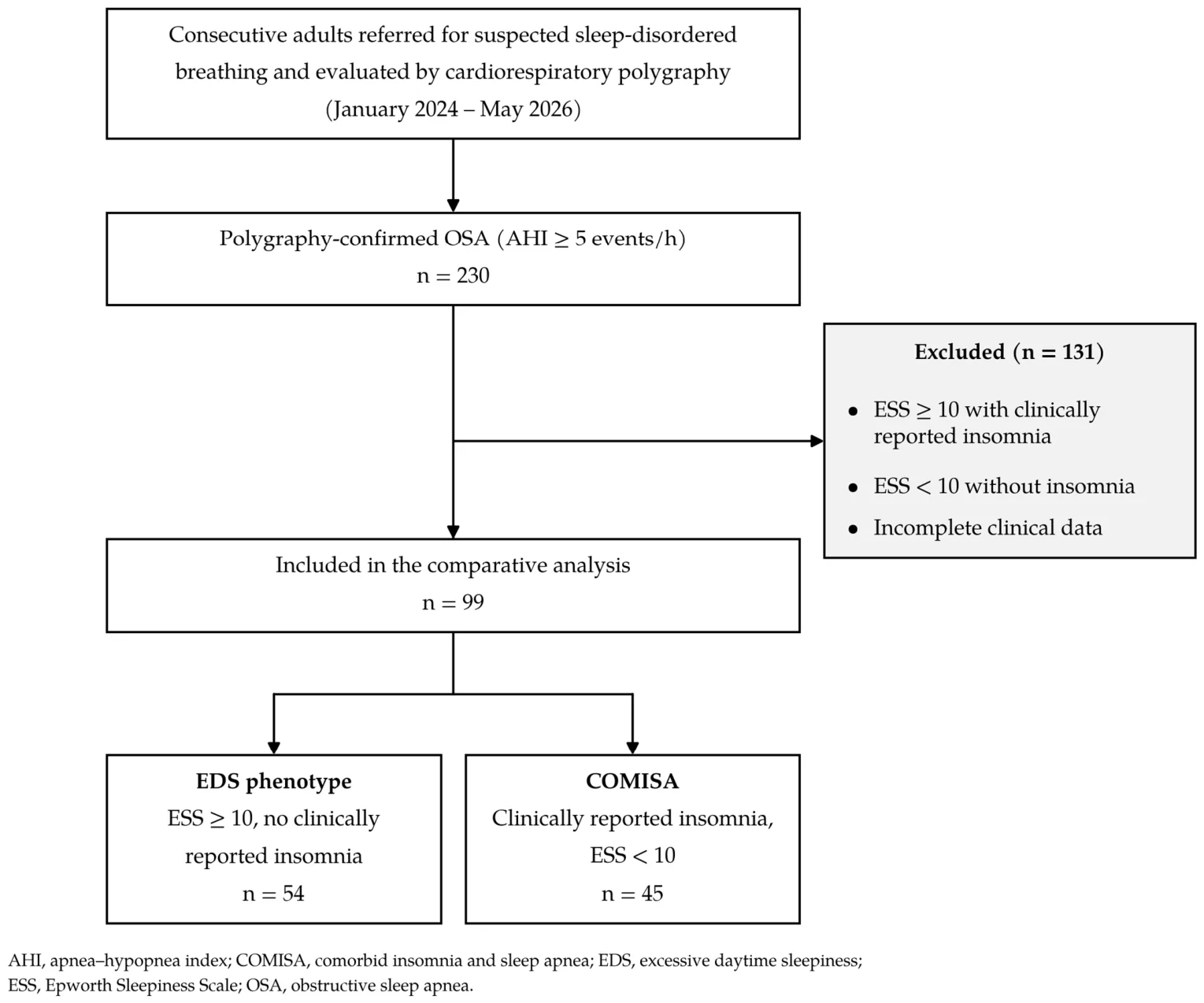
Patients recruitment flowchart.

We conducted a retrospective cross-sectional analysis at a single center, the “Leon Daniello” Clinical Hospital of Pulmonology in Cluj-Napoca, Romania. Consecutive adults referred for suspected sleep-disordered breathing between January 2024 and May 2026 were included. All patients were evaluated by cardiorespiratory polygraphy at the sleep laboratory as part of their standard diagnostic work-up. The polygraph used was Nox T3. It’s a home sleep testing device that records the flux (nasal canula), respiratory effort (2 bands one on the thorax and one on the abdomen) and a puls oximeter (for the heart rate and for the saturation). The software used for the interpretation was Noxturnal T3 version 2.6. Every polygraphy was revised by the same pulmonologist. No study-specific procedures or interventions were performed. The data were extracted from the existing clinical records and sleep-laboratory database after the approval of the Ethics Committee of the “Iuliu Hațieganu” University of Medicine and Pharmacy (protocol no. 172, 17 June 2026). This approval covers the retrospective use of anonymized data from patients evaluated during the entire period, starting from January 2024. All patients had given written informed consent at the time of their hospital evaluation, which included consent for the use of their clinical data for research purposes. OSA was diagnosed according to the American Academy of Sleep Medicine criteria [28] and defined as an AHI ≥ 5 events/h. Of the 230 patients with confirmed OSA, those who met the criteria for one of the two phenotypes defined below and had complete clinical data were included in the comparative analysis (*n* = 99). The patients’ recruitment process is shown in Figure 1.

### 2.2. Phenotype Definitions

Daytime sleepiness was assessed with the Epworth Sleepiness Scale (ESS) [29]. Insomnia was ascertained clinically during the standardized pre-polygraphy consultation by a sleep physician, who recorded: difficulty initiating sleep, difficulty maintaining sleep and/or early morning awakening, their frequency and duration, associated daytime impairment, and current hypnotic use. Insomnia was classified as clinically reported when the patients reported one of the above symptoms. These elements correspond to the core ICSD-3 criteria for chronic insomnia disorder, but no validated insomnia questionnaire was administered. The term ‘COMISA’ in this study therefore refers to OSA with clinically significant insomnia complaints. The excessive daytime sleepiness phenotype (EDS) was defined as ESS ≥ 10 in the absence of clinically reported insomnia. COMISA was defined as OSA accompanied by clinically reported insomnia with an ESS score < 10. The two groups were mutually exclusive by design.

### 2.3. Sleep Study and Clinical Variables

Polygraphic variables included the apnee-hypopneea index (AHI), the oxygen desaturation index (ODI), and nadir and mean oxygen saturation (SpO_2_). The STOP-BANG score, smoking status and body mass index (BMI) were recorded.

### 2.4. Cardiovascular and Metabolic Comorbidity

Cardiovascular comorbidities were recorded from the clinical record: arterial hypertension, cardiac arrhythmias, ischemic heart disease, heart failure, cor pulmonale/pulmonary hypertension, and stroke. Type 2 diabetes mellitus and dyslipidemia were recorded as metabolic comorbidities. A composite cardiovascular burden score (range 0–6) was computed as the number of these six cardiovascular conditions present in each patient, and “any cardiovascular disease” was defined as the presence of at least one of them. The score was intended as a simple measure of the number of coexisting cardiovascular diagnoses rather than an index of disease severity. Equal weights were assigned because no validated weighting system for these conditions is available in OSA populations, and an unweighted count avoids arbitrary weighting and is readily reproducible.

### 2.5. Statistical Analysis

Continuous variables are presented as mean ± standard deviation (SD) and compared with the independent-samples *t*-test, or as median (25th–75th percentile) and compared with the Mann–Whitney U test. Categorical variables are presented as counts (percentages) and compared with the χ^2^ test or the z-test for proportions. A multivariable binary logistic regression model was fitted with phenotype as the dependent variable (COMISA vs. EDS, with EDS as reference). Age, sex, BMI, dyslipidemia, cardiac arrhythmias, arterial hypertension and type 2 diabetes mellitus were entered simultaneously as independent variables. Adjusted odds ratios (aOR) with 95% confidence intervals (CI) are reported. Model performance was assessed with the omnibus test of model coefficients, Nagelkerke R^2^, the Hosmer–Lemeshow goodness-of-fit test and the classification table at a 0.50 cut-off. A two-sided *p*-value < 0.05 was considered statistically significant. Analyses were performed with IBM SPSS Statistics, version 25 (IBM Corp., Armonk, NY, USA). Because of the retrospective design, no a priori sample-size calculation was performed. The prevalence of any cardiovascular disease and the cardiovascular burden score were prespecified as primary comparisons; all other comparisons were considered exploratory.

A generative AI tool (Claude, Anthropic) (Claude Opus 5.5, Anthropic) was used to support manuscript preparation: language editing; checking bibliographic details, citation order and MDPI reference formatting; drafting text on the statistical strategy and generating the plotting code used to draw Figure 1 and the patient-selection flow chart from the authors’ data. The AI tool was not used to collect, analyze or interpret data. All statistical analyses were performed by the authors in IBM SPSS Statistics, and all AI-assisted text and figures were checked by the authors against the original data.

## 3. Results

### 3.1. Cohort Characteristics

Of the 230 patients with OSA, 54 met the criteria for the excessive daytime sleepiness phenotype and 45 for COMISA and had complete data. The two groups were similar in age and sex distribution (Table 1). As expected from the phenotype definitions, ESS scores were substantially higher in the EDS group (14.06 ± 3.10 vs. 7.62 ± 5.84, *p* < 0.001).

OSA severity did not differ significantly between groups. The AHI was numerically higher in the EDS group (42.22 vs. 37.78 events/h, *p* = 0.328), whereas the ODI was numerically higher in the COMISA group (37.20 vs. 34.12 events/h, *p* = 0.492). Nocturnal oxygenation was comparable, with similar nadir SpO_2_ (median 79% vs. 77%, *p* = 0.630) and mean SpO_2_ (median 91% in both groups, *p* = 0.784).

### 3.2. Cardiovascular and Metabolic Comorbidity

**Figure 2:**
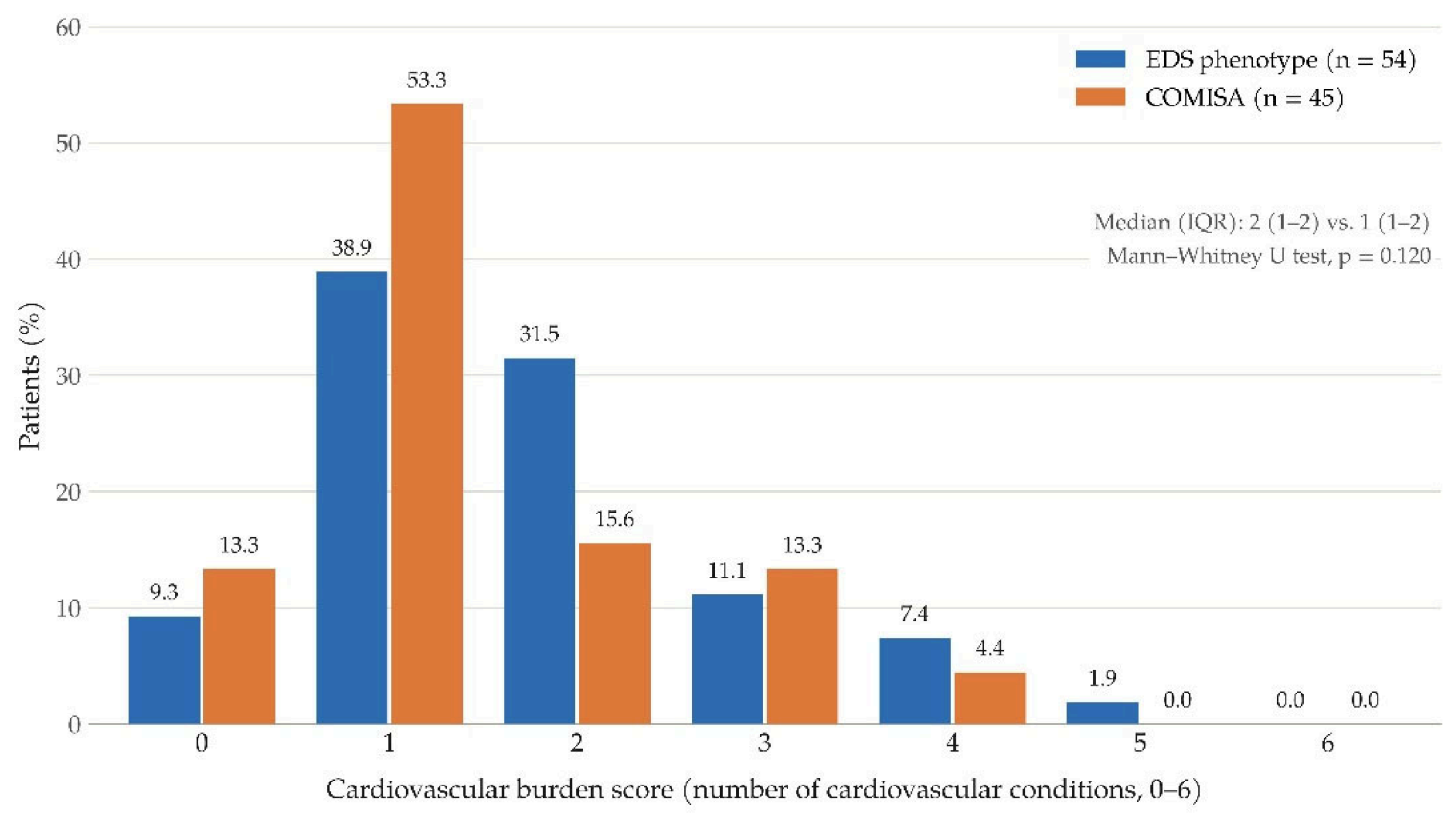
Distribution of the cardiovascular burden score (number of cardiovascular conditions, range 0–6) in the excessively sleepy (EDS) and COMISA phenotypes. Values above bars are percentages of patients in each group.

The prevalence of any cardiovascular disease was high and comparable between groups (90.7% vs. 86.7%, *p* = 0.521), as was the cardiovascular burden score (Table 2). The burden score was numerically higher in the EDS group (1.74 ± 1.13 vs. 1.42 ± 1.03; median 2 vs. 1), but the difference was not statistically significant (*p* = 0.120) (Figure 1). Arterial hypertension was the dominant comorbidity, present in roughly four of five patients in both groups. Heart failure (35.2% vs. 20.0%, *p* = 0.095) and cor pulmonale/pulmonary hypertension (16.7% vs. 8.9%, *p* = 0.254) were each numerically more frequent in the EDS group, although none reached statistical significance. Ischemic heart disease and stroke were similarly prevalent in both groups. There is an arrhythmic tendency in both groups (20.4% vs. 6.7%, *p* = 0.051)(see Figure 2).

Regarding metabolic comorbidity, patients in the EDS group had a significantly higher mean BMI (38.59 vs. 35.35 kg/m^2^, *p* = 0.020) and twice the frequency of class III obesity (44.4% vs. 22.2%, *p* = 0.020). Dyslipidemia seems to be more frequent in COMISA patients (64.4% vs. 44.4%, *p* = 0.047). Type 2 diabetes mellitus was numerically more frequent in the EDS group (35.2% vs. 22.2%, *p* = 0.158).

### 3.3. Multivariable Model

The multivariable logistic regression model was statistically significant compared with the intercept-only model (omnibus test, *p* = 0.011) and had modest explanatory power (Nagelkerke R^2^ = 0.225). The Hosmer–Lemeshow test did not indicate lack of fit (*p* = 0.838). At a classification threshold of 0.50, the model correctly classified 70.4% of EDS patients and 64.4% of COMISA patients, for an overall accuracy of 67.7%.

Three variables were independently associated with phenotype (Table 3). Higher BMI was associated with lower odds of COMISA, with each 1 kg/m^2^ increase corresponding to an approximately 8% reduction in odds (aOR = 0.920; 95% CI: 0.859–0.986; *p* = 0.019). Dyslipidemia was associated with approximately 2.5-fold higher odds of COMISA (aOR = 2.508; 95% CI: 1.007–6.247; *p* = 0.048). Cardiac arrhythmias were associated with lower odds of COMISA (aOR = 0.203; 95% CI: 0.044–0.947; *p* = 0.042), corresponding to approximately 4.9-fold higher odds of the EDS phenotype. Age, sex, arterial hypertension and type 2 diabetes mellitus were not independently associated with phenotype. The estimates for dyslipidemia and arrhythmias should be interpreted cautiously, given *p*-values close to 0.05 and wide confidence intervals.

## 4. Discussion

In this single-center comparison of two symptomatic OSA phenotypes, cardiovascular comorbidities were highly prevalent and broadly comparable between patients with excessive daytime sleepiness (EDS) and those with comorbid insomnia and sleep apnea (COMISA). Differences seem more apparent in metabolic characteristics. Patients with the EDS clinical phenotype had higher BMI and approximately twice the prevalence of class III obesity, alongside numerically higher frequencies of arrhythmias, heart failure, and cor pulmonale, whereas dyslipidemia seemed to be more frequent in COMISA. Exploratory multivariable logistic regression showed that higher BMI and arrhythmias were associated with greater odds of EDS, while dyslipidemia was associated with COMISA, after adjustment for other covariates. However, the small sample size and limited events-per-variable ratio increase the risk of overfitting and unstable estimates, warranting cautious interpretation. The model’s limited classification performance and the broadly similar cardiovascular profiles suggest considerable phenotypic overlap. These findings remain exploratory and require validation in larger cohorts.

### 4.1. Both Phenotypes Carry a High Cardiovascular Burden

About nine in ten patients in each group had at least one cardiovascular condition, and roughly 80% had hypertension. This is consistent with the well-established association between OSA and hypertension, coronary disease and heart failure [1,2,30,31]. Each phenotype has also been linked to adverse outcomes when compared with other OSA patients. In cluster-analytic studies, the EDS phenotype carried the highest risk of incident cardiovascular disease [7,8], and this risk was amplified in combination with a high hypoxic burden [9]. COMISA has likewise been associated with more prevalent and incident cardiovascular disease, cerebrovascular events and mortality in community, clinical and veteran cohorts [17,18,19,20], and recent registry and older-adult cohort data have further examined the COMISA–cardiovascular relationship [32,33]. The present study tries to compare the cardiovascular and metabolic comorbidities between the two groups. Most previous studies compared each phenotype with non-EDS phenotype or insomnia-free OSA. When the two high-risk symptom profiles are compared with each other, the difference in overall cardiovascular burden is small, which indicates that the choice of comparator strongly shapes conclusions about phenotype-specific risk. Referral to a tertiary sleep laboratory has probably also enriched both groups for cardiometabolic disease.

### 4.2. The EDS Phenotype

The trends toward more arrhythmias, heart failure and cor pulmonale in the EDS group agree with Mazzotti et al., who found the highest incidence of heart failure and cardiovascular events in the excessively sleepy subtype [8], and with the higher incidence of coronary calcification in sleepy OSA patients in ELSA-Brasil [10]. In our cohort, the AHI, ODI and nadir SpO_2_ were similar across groups, so these trends are unlikely to be explained by conventional severity metrics. Hypoxic burden, not evaluated in our study may predict cardiovascular mortality better than the AHI [14,15,16] and may differ between phenotypes even when the AHI does not. Arrhythmias in OSA are promoted by intermittent hypoxia, large intrathoracic pressure swings, sympathetic surges and progressive atrial remodeling [11,13,34,35]. These mechanisms are likely to be amplified by the severe obesity that generally characterized EDS group, so obesity may be both a determinant and a partial mediator of risk in this phenotype. Central obesity has recently been associated with recurrent cardiovascular events in the SAVE population [36]. Sleepiness itself may also signal systemic inflammation and metabolic dysregulation rather than simply reflecting sleep fragmentation [3,4]. Finally, objective sleepiness appears to predict mortality more strongly than ESS-based sleepiness [37], so subjective classification may underestimate the risk carried by truly sleepy patients.

### 4.3. Dyslipidemia in Comisa

The slightly higher prevalence of dyslipidemia in COMISA was unexpected, particularly given the lower BMI in this group. It contrasts with recent ESADA data, in which excessive sleepiness, but not insomnia, was associated with dyslipidemia in OSA [25]. Several explanations are possible. First, intermittent hypoxemia and sleep fragmentation are each independently associated with dyslipidemia in OSA [38,39,40]; COMISA patients in our cohort had a numerically higher ODI, and their fragmented, shortened sleep may add a further contribution. Second, chronic hyperarousal, hypothalamic–pituitary–adrenal axis activation and stress-related neural activity have been linked to cardiometabolic risk factors and vascular events [24,41]. Third, detection or treatment bias is possible: COMISA patients may have more frequent medical contact and lipid testing, and treated dyslipidemia recorded as a diagnosis cannot be distinguished from untreated disease in our data. Fourth: COMISA definitions: Insomnia definition in COMISA research vary widely, from symptom-level definitions (e.g., chronic difficulty initiating or maintaining sleep) to disorder-level ICSD-3 criteria or ISI cut-offs, most often ≥15. These definitions identify populations that differ in prevalence and associated morbidity [42,43,44]. Large recent clinical datasets have likewise relied on routinely collected self-report insomnia items rather than formal diagnostic criteria [32]. Our approach is therefore comparable to real-world clinical phenotyping, but the “COMISA” label in our study most closely reflects clinically significant insomnia symptoms rather than a confirmed insomnia disorder. We have revised the terminology throughout to make this clear.

Regarding classification, we consider two sources of error:

False-positive COMISA: Patients with milder or short-term insomnia symptoms may have been classified as COMISA, which would make the COMISA group more heterogeneous.

False-negative COMISA: Undocumented insomnia in patients with ESS ≥ 10 may have led to some COMISA patients being classified into the EDS group. This concern is relevant because subjective sleepiness can co-exist with insomnia and may dominate the clinical consultation [27].

Finally, the ESADA analysis used a much larger sample and a different insomnia definition, and the lower confidence limit of our estimate (1.007) lies very close to unity. This finding should therefore be considered hypothesis-generating and requires confirmation with measured lipid profiles and medication data.

### 4.4. Pathophysiological and Clinical Implications

COMISA is increasingly recognized as a distinct entity, with a bidirectional relationship between its components [6], an OSA endotype that differs from OSA alone [21].

Our results suggest that the cardiovascular profile of COMISA is not milder than that of sEDS-OSA but differently composed: less driven by obesity and possibly more metabolic. Clinically, both phenotypes warrant systematic cardiometabolic screening. In sleepysymptometic (EDS), severely obese patients, attention to rhythm disturbances and heart failure appears justified. In COMISA, lipid assessment and combined management of both disorders, such as cognitive behavioral therapy for insomnia alongside positive airway pressure, may be relevant [27,45]. Whether phenotype-guided management modifies cardiovascular outcomes remains an open question [26].

### 4.5. Strengths and Limitations

The main strengths of this study include the direct comparison of two clinically recognizable phenotypes within the same study population, and the simultaneous assessment of cardiovascular and metabolic comorbidities.

Several limitations should be acknowledged. First, the cross-sectional design precludes conclusions regarding causality or temporal relationships. Second, the relatively small sample size and single-center setting limit statistical power and the generalizability of the findings. Some observed differences were of borderline statistical significance, and no adjustment for multiple comparisons was performed, increasing the possibility of chance findings.

Importantly, the multivariable logistic regression analysis included seven predictors in a sample of 99 participants, including 45 patients with COMISA, resulting in an events-per-variable ratio of approximately 6.4, assuming one parameter per predictor. Although the metabolic and cardiovascular variables were selected based on their clinical and pathophysiological relevance, the limited number of events relative to model complexity may have increased the risk of overfitting, coefficient instability, and imprecise effect estimates. Furthermore, the relatively low frequency of certain cardiovascular conditions, particularly ischemic heart disease, heart failure and stroke, limited the statistical information available for evaluating their individual associations. Consequently, even statistically significant associations identified in the regression analysis should be interpreted cautiously and regarded as exploratory rather than definitive. The model also misclassified nearly one-third of participants and should not be considered suitable for individual clinical prediction without further validation.

Additionally, insomnia was identified through clinical assessment rather than a validated instrument such as the Insomnia Severity Index, while daytime sleepiness was assessed using subjective measures. Information on comorbidities was obtained from medical records, without detailed data on disease severity, lipid profiles, or medication use. Measures of hypoxic burden and other potentially relevant physiological parameters were also unavailable. The absence of a comparison group consisting of patients with OSA without either insomnia or excessive daytime sleepiness limited the characterization of phenotype-specific associations.

Insomnia was ascertained from the clinical consultation rather than with a validated instrument such as the Insomnia Severity Index, and the documented elements were not captured on a standardized form. Because COMISA definitions based on symptoms, questionnaire cut-offs and formal ICSD-3 criteria identify different populations, our COMISA group may be more heterogeneous than in studies using standardized instruments. Some patients with undocumented insomnia may also have been classified in the EDS group. Non-differential misclassification would be expected to attenuate between-phenotype differences. Differential documentation, however, for example, in patients with greater medical contact, cannot be excluded and may have contributed to the observed association with dyslipidemia. In addition, by requiring an ESS below the sleepiness threshold, our COMISA group represents a predominantly non-sleepy COMISA subtype, and the findings may not apply to COMISA patients with concomitant excessive sleepiness. Future prospective, multicenter studies involving larger patient cohorts, validated sleep assessments, objective measures of daytime sleepiness, hypoxic burden, and longitudinal cardiovascular outcomes are warranted to confirm these findings and determine whether the investigated phenotypes are associated with different cardiovascular and metabolic risk profiles.

## 5. Conclusions

In patients with confirmed OSA, the excessive daytime sleepiness and COMISA phenotype showed a similarly high overall prevalence of cardiovascular disease but distinct metabolic profiles: greater adiposity and an arrhythmic tendency in the sleepy phenotype, and an independent association with dyslipidemia in COMISA. These hypothesis-generating findings support symptom-based phenotyping as a simple tool for targeting cardiometabolic assessment in OSA and should be tested prospectively.

## Figures and Tables

**Table 1:** Demographic, anthropometric and sleep characteristics of the two phenotype groups.

Characteristic	EDS Phenotype (*n* = 54)	COMISA (*n* = 45)	*p*
Age, years	58.78 ± 11.00	60.60 ± 12.37	0.440
Male sex	39 (72.2)	28 (62.2)	0.289
Current smoker	16 (29.6)	10 (22.2)	0.635
Never smoker	31 (57.4)	22 (48.9)	0.397
ESS score	14.06 ± 3.10 13 (11.75–16)	7.62 ± 5.84 7 (3–9.5)	<0.001
STOP-BANG score	6 (5–7)	6 (4.5–7)	0.291
AHI, events/h	42.22 ± 23.70	37.78 ± 20.55	0.328
ODI, events/h	34.12 ± 22.93	37.20 ± 21.09	0.492
Nadir SpO_2_, %	75.03 ± 10.94 79 (72–82)	73.95 ± 11.46 77 (66.5–82)	0.630
Mean SpO_2_, %	90.53 ± 3.20 91 (89–92.93)	89.59 ± 5.38 91 (87.95–93.15)	0.784

Continuous data are shown as mean ± SD (independent-samples *t*-test) and/or median (25th–75th percentile) (Mann–Whitney U test); categorical data as *n* (%) (χ^2^ test) AHI, apnea–hypopnea index; COMISA, comorbid insomnia and sleep apnea; EDS, excessive daytime sleepiness; ESS, Epworth Sleepiness Scale; ODI, oxygen desaturation index; SpO_2_, peripheral oxygen saturation.

**Table 2:** Prevalent cardiovascular and metabolic comorbidity by phenotype.

Comorbidity	EDS Phenotype (*n* = 54)	COMISA (*n* = 45)	*p*
Any cardiovascular disease	49 (90.7)	39 (86.7)	0.521
Cardiovascular burden score (0–6)	1.74 ± 1.13 2 (1–2)	1.42 ± 1.03 1 (1–2)	0.120
Arterial hypertension	43 (79.6)	38 (84.4)	0.536
Cor pulmonale/pulmonary hypertension	9 (16.7)	4 (8.9)	0.254
Cardiac arrhythmias	11 (20.4)	3 (6.7)	0.051
Ischemic heart disease	9 (16.7)	7 (15.6)	0.881
Heart failure	19 (35.2)	9 (20.0)	0.095
Stroke	3 (5.6)	3 (6.7)	0.818
BMI, kg/m^2^	38.59 ± 11.00	35.35 ± 6.42	0.020
Class III obesity (BMI ≥ 40 kg/m^2^)	24 (44.4)	10 (22.2)	0.020 †
Type 2 diabetes mellitus	19 (35.2)	10 (22.2)	0.158
Dyslipidemia	24 (44.4)	29 (64.4)	0.047

Categorical data are shown as *n* (%) (χ^2^ test); continuous data as mean ± SD (independent-samples *t*-test) and/or median (25th–75th percentile) (Mann–Whitney U test). † z-test for proportions. BMI, body mass index; COMISA, comorbid insomnia and sleep apnea; EDS, excessive daytime sleepiness.

**Table 3:** Multivariable binary logistic regression for COMISA versus the excessively sleepy phenotype (reference) (*n* = 99).

Variable	aOR	95% CI	*p*
Age (per year)	1.029	0.984–1.075	0.208
Sex (male vs. female)	0.751	0.287–1.970	0.561
BMI (per kg/m^2^)	0.920	0.859–0.986	0.019
Dyslipidemia	2.508	1.007–6.247	0.048
Cardiac arrhythmias	0.203	0.044–0.947	0.042
Arterial hypertension	0.874	0.239–3.191	0.838
Type 2 diabetes mellitus	0.590	0.215–1.620	0.306

All variables were entered simultaneously. Omnibus test *p* = 0.011; Nagelkerke R^2^ = 0.225; Hosmer–Lemeshow *p* = 0.838; overall correct classification 67.7%. aOR, adjusted odds ratio; BMI, body mass index; CI, confidence interval; COMISA, comorbid insomnia and sleep apnea.

## Data Availability

The data presented in this study are available on request from the corresponding author due to patient privacy restrictions.

## Abbreviations

The following abbreviations are used in this manuscript:

AHI	apnea–hypopnea index
aOR	adjusted odds ratio
BMI	body mass index
CI	confidence interval
COMISA	comorbid insomnia and sleep apnea
EDS	excessive daytime sleepiness
ESS	Epworth Sleepiness Scale
ODI	oxygen desaturation index
OSA	obstructive sleep apnea
SD	standard deviation
SpO_2_	peripheral oxygen saturation
